# Supplementary material for: Study protocol: Minimum effective low dose: anti-human thymocyte globulin (MELD-ATG): phase II, dose ranging, efficacy study of antithymocyte globulin (ATG) within 6 weeks of diagnosis of type 1 diabetes
Source: BMJ Open. 2021 Dec 6;11(12):e053669. doi: 10.1136/bmjopen-2021-053669 (PMC8655536; doi:10.1136/bmjopen-2021-053669)
Supplement: Supplementary data [file bmjopen-2021-053669supp001.pdf]

Supplementary information-MELD-ATG Protocol appendix

Legal status of the investigational medical product (IMP)/placebo:

ATG is a pasteurised rabbit polyclonal antibody specific for human thymocytes. It is an immunosuppressive agent, indicated and marketed under the trade name Thymoglobuline® for several indications. ATG is currently registered or authorised for sale in more than 60 countries worldwide. In each country planned to participate in MELD-ATG, indications that ATG is approved for varies, but include: 1) prevention of transplant/graft rejection (renal, heart, liver, pancreas, and other), 2) treatment of transplant/graft rejection (renal, heart, liver, pancreas, and other), 3) aplastic anaemia, 4) prevention and treatment of GvHD[22]. Within this trial, ATG is being used as IMP in an unlicensed indication (newly diagnosed T1D), and at total doses (0.1 – 2.5 mg/kg total dose) lower than the total doses used for the above licenced indications; however, it will be administered by the licensed route (IV). The trial is being carried out under a Clinical Trial Authorisation. The drug is therefore only to be used by the named investigators, for participants specified in this protocol, and within the trial.

Supply of IMP/Placebo:

Thymoglobuline® will be supplied by Sanofi according to good manufacturing practices and local regulatory specifications and requirement. The IMP will be supplied as single-use 10-ml vials containing 25mg of ATG sterile lyophilised powder, intended for reconstitution prior to IV administration according to the manufacturer instructions.

ATG vials will be supplied to site pharmacies with trial-specific labels applied. Site pharmacies will be unblinded to treatment allocation to allow for preparation of required infusions. Infusions for both ATG and placebo will be packaged and labelled in a similar manner in order to maintain the blind for participants and the site research team. Pharmacies will be responsible for applying the trial labelling to ATG (and placebo) infusion bags and participant-specific details will be added to the labelling prior to dispensing.

Accountability of the trial treatment:

All pharmacy aspects of the trial at the participating site are the responsibility of the Principal Investigator (PI) who will delegate this responsibility to the local pharmacist, or other appropriately qualified personnel. This delegation of duties must be recorded on the delegation log. The PI or the delegated individual (e.g. the trial pharmacist), must ensure that the trial medications are stored and dispensed in accordance with local practice, applicable regulatory requirements and trial specific prescriptions.

Pharmacy will be unblinded to the randomisation allocation via a concealment list, in order to prepare the appropriate ATG/placebo infusions. The unblinded pharmacy staff will keep the treatment information confidential and will not discuss or release information on treatment allocation. The placebo is 0.9% sodium chloride solution and will be sourced from local hospital stock. Placebo treatment will be prepared to be identical in volume and appearance as the ATG infusion bag. The premedication given to manage the expected adverse reactions to infusion of ATG will be sourced from local hospital stock and are the responsibility of pharmacy.

ATG/placebo in 500ml sodium chloride 0.9% intravenous infusion bag will be dispensed by the pharmacy at the participating site in accordance with trial-specific prescription. A prescription template will be provided, although sites will be permitted to use their own clinical trial template prescription if suitable. The prescription will not state the dose of the ATG to be prepared. The

pharmacy will be responsible for calculating the dose according to the randomisation and the cohort that the patient has been allocated via the electronic system. The dose will not be added on the label as this would unblind the patient and administering staff.

For ATG, sites will maintain a full accountability record documenting receipt, storage, dispensing, administration, return of unused ATG to the pharmacy, and used and unused ATG packaging as appropriate, as specified in the MELD-ATG Pharmacy Manual. For placebo, accountability records will be as per local standard accountability procedures. Template accountability forms will be supplied; however, sites are permitted to use their own drug accountability forms as long as the same information is recorded and is available to the Sponsor. At the end of the trial, it must be possible to reconcile ATG supply and usage of stock. Account must be given of any discrepancies and documents of delivery. Sponsor authorisation will be required prior to destruction of returned or expired ATG. Returned placebo will be documented to allow verification of treatment compliance; however, Sponsor authorisation will not be required prior to destruction. All unopened and or unused vials of ATG may be destroyed as per institutional guidelines upon completion of the trial or if the drug expires, upon approval of the sponsor. Template destruction forms will be available and will be supplied as part of the PSF.

When participants are randomised, they will be allocated a dose of ATG, or placebo, within the cohort. The unblinded personnel (e.g. trial pharmacist) will be responsible for calculating the dose for the patient. The actual weight of the participant at the baseline visit will be used to calculate the dose.

Definitions for assessment of safety in MELD-ATG: Safety will be assessed by frequency, incidence and nature of adverse events (AEs) and serious adverse events (SAEs) arising during the study. An AE is any untoward medical occurrence in a participant or clinical trial participant administered a medicinal product and which does not necessarily have a causal relationship with this treatment. An AE can therefore be any unfavourable and unintended sign (including an abnormal laboratory finding), symptom, or disease temporally associated with the use of the IMP, whether or not considered related to the IMP. Recording of all AEs must start from the point of written informed consent/assent, regardless of whether a participant has yet received a medicinal product.

An adverse reaction to an investigational medicinal product (AR) is an untoward and unintended responses to the IMP related to any dose administered. All AEs judged by either the reporting investigator or the Sponsor as having a reasonable causal relationship to a medicinal product qualify as ARs. The expression reasonable causal relationship means to convey in general that there is evidence or argument to suggest a causal relationship.

An unexpected AR is an adverse reaction whose nature or severity is not consistent with the applicable reference safety information (RSI). When the outcome of the AR is not consistent with the applicable RSI this AR should be considered as unexpected. The term “severe” is often used to describe the intensity (severity) of a specific event. This is not the same as “serious,” which is based on participant /event outcome or action criteria.

A serious adverse event or serious adverse reaction (SAE/SAR) is any untoward medical occurrence that at any dose: 1) results in death, 2) is life-threatening, 3) requires hospitalisation or prolongation of existing inpatients hospitalisation, 4) results in persistent or significant disability or incapacity, 5) is a congenital anomaly or birth defect or 6) is an important medical event - some medical events may jeopardise the participant or may require an intervention to prevent one of the above characteristics/ consequences. Such events (hereinafter referred to as ‘important medical events’)

should also be considered as “serious”. “Life-threatening” in the definition of a SAE or SAR refers to an event in which the participant was at risk of death at the time of event; it does not refer to an event which hypothetically might have caused death if it were more severe.

A suspected unexpected serious adverse reaction (SUSAR) is a SAR, the nature and severity of which is not consistent with the information set out in the RSI.

Reference safety information (RSI) is a list of medical events that defines which reactions are expected for the IMP within a given trial and thus determining which SARs require expedited reporting. Expected adverse reactions/serious adverse reactions (AR /SARs) are listed in the latest regulatory authority-approved version of the RSI. This must be used when determining the expectedness of the AR. If the AR meets the criteria for seriousness, this must be reported.

Study Samples:

MELD-ATG will utilise the INNODIA consortium established SOPs for sample collection, shipments, storage and analysis. All MELD-ATG sample shipments from participating sites to central laboratories will be recorded via the eCRF. Samples sent to central laboratories will be pseudo-anonymised. The following central INNODIA consortium laboratories will be used for analysis and storage of MELD-ATG biological samples: 1) Core Biochemical Assay Laboratory (CBAL) at the Cambridge University Hospitals NHS Foundation Trust, UK led by Mr Keith Burling, 2) Paediatric Diabetes Research Group (PEDIA) Laboratory at the University of Helsinki, Helsinki, Finland led by Prof Mikael Knip, 3) Peter Gorer Department of Immunobiology at the School of Immunology and Microbial Sciences, King’s College London, Guy’s Hospital, London, UK led by Dr Tim Tree, 4) Diabetes and Autoimmunity Research (DeAR) Lab at the Institut Cochin, INSERM U1016, Paris, France led by Prof Roberto Mallone, 5) Diabetes Research Lab at the Leiden University Medical Centre, Leiden, The Netherlands led by Prof Bart Roep, 6) Centre for Regenerative Therapies Dresden at the Technische Universität Dresden, Dresden, Germany led by Prof Ezio Bonifacio, 7) Department of Medical Sciences, Surgery and Neurosciences at the University of Siena, Siena, Italy led by Dr Guido Sebastiani, 8) JDRF/Wellcome Trust Diabetes and Inflammation Laboratory at the University of Oxford, Oxford, UK led by Prof John Todd with 2)-7) being INNODIA immune hubs. The MELD-ATG Trial Manual details sample collection, processing and shipment requirements in more detail.

All other samples collected for exploratory studies (blood, urine and stool for Omics analysis) will be processed locally at participating sites according to the respective INNODIA SOPs and stored locally in <-69°C in freezers with temperature monitoring until shipped in batches to the University of Cambridge. Until required for exploratory analysis, samples will be sorted and stored by the University of Cambridge, at the University of Cambridge or at the National Institute of Health Research National Biosample Centre. When requested, samples will be sent to selected analytical laboratories. At every location, samples will be kept at <-69°C in freezers with temperature monitoring. For transport, samples will be shipped on dry ice. All shipments will be recorded in the INNODIA data warehouse which tracks the location of the samples.

Toxicology and normal standard of care samples:

Samples collected in the trial that are part of the normal standard of care or for screening/safety (for example HbA1c, FBC, biochemistry, HIV, hepatitis, EBV and SARS-CoV-2 serology and PCR) will be analysed locally by the local hospital accredited laboratory and samples discarded once analysed as per normal local protocols.

Mixed Meal Tolerance Test (MMTT) measurements:

Wilhelm-Benartzi CS, *et al. BMJ Open* 2021; 11:e053669. doi: 10.1136/bmjopen-2021-053669

At baseline and at 3, 6 and 12 months trial participants will have an initial 120-minute MMTT, during which blood will be collected for measuring glucose and C-peptide. Glucose levels should be assessed prior to ingestion of the liquid meal. The MMTT will only be performed if the glucose level (as assed at point of care) is between 4 and 11.1 mmol/l.

Long-acting insulin or basal rates (in case of using an insulin pump) use may be continued throughout the MMTT, rapid-acting insulin use is acceptable up to 2 hours before the MMTT to correct hyperglycaemia and short-acting insulin use is acceptable up to 6 hours before the MMTT to correct hyperglycaemia.

For the MMTT, glucose will be measured by the local hospital laboratory; C-peptide will be measured centrally. Fasted participants will be given a fixed liquid meal (dose based on baseline body weight). The meal solution needs to be taken orally and ingested within 10 minutes. Blood samples for the measurement of glucose and C-peptide should be collected at approximately 10 minutes prior to the meal (-10 min), immediately before ingestion (0 minutes), and 15, 30, 60, 90 and 120 minutes after start of ingestion.

Dried Blood Spot (DBS) measurements:

Fasted participants will be requested to collect DBS samples and record capillary blood glucose measurements at home, starting in the first 4 weeks after completing trial treatment and continuing monthly (every 4 weeks ± 1 week) until end of trial participation (12 months post treatment). DBS samples and corresponding blood glucose measurements will be taken immediately before ingestion of a liquid meal and after 60 minutes from the start of ingestion, by finger prick in the home setting. The DBS liquid meal should provide the same nutrients per kg of body weight as the MMTT liquid meal. Fasted participants will be asked to withhold their pre-breakfast insulin until after the post-prandial DBS has been taken so as not to interfere with the C-peptide result. During the DBS collection, short or rapid-acting insulin should not be used until the end of the collection (60 minutes post ingestion of liquid meal). Following the 60-minute DBS sample and blood glucose measurement, the participant will give a correction dose of insulin either via injection or pump, according the participant's own insulin sensitivity factor. Baseline DBS collection and capillary blood glucose (and ketones, as indicated by glucose) measurements will be performed in parallel with the baseline MMTT. For glucose >8 mmol/l, a subcutaneous insulin correction dose to be given, either via injection or pump, according to the participant's own insulin sensitivity factor but a ketones test not required. For glucose >14 mmol/l, ketones are to be tested by finger prick and for ketones >0.6 mmol/l, glucose and ketones to be repeated until ketones have decreased <0.6 mmol/l.

Wilhelm-Benartzi CS, *et al. BMJ Open* 2021; 11:e053669. doi: 10.1136/bmjopen-2021-053669
